# The efficacy and safety of intravenous tranexamic acid in patients with posterior operation of multilevel thoracic spine stenosis: a prospective randomized controlled trial

**DOI:** 10.1186/s12891-022-05361-2

**Published:** 2022-05-02

**Authors:** Tan Lei, Wen Bingtao, Guo Zhaoqing, Chen Zhongqiang, Liu Xin

**Affiliations:** Department of Orthopaedics, Peking University International Hospital, Life Park Road No 1 Life Science Park of Zhong Guancun, Changping District, Beijing, 102206 China

**Keywords:** Tranexamic acid (TXA), Perioperative blood loss, Complication, Thoracic spine

## Abstract

**Background:**

This study was a randomized controlled trial to evaluate efficacy and safety of the usage of intravenous tranexamic acid during posterior operation of multilevel thoracic spine stenosis for controlling perioperative blood loss.

**Methods:**

Sixty eight patients with multilevel thoracic spine stenosis were randomized into the tranexamic acid group receiving 15 mg/kg body weight before the skin incision was made and 1 mg/kg body weight per hour during operation or the control group receiving the same dose of placebo (0.9% sodium chloride solution) intravenously. Pedicle screw fixation, laminectomy and selective discectomy were performed. Intraoperative and perioperative total blood loss were compared. The necessity and amount for blood transfusion, blood coagulation function, durations of postoperative hospital stays were compared. The complications of tranexamic acid were also investigated such as cardiovascular and cerebrovascular events, lower limb venous thrombosis.

**Results:**

There were no statistically significant differences in age, gender, body mass index, ASA status, pathology required surgery, preoperative hemoglobin, operation time, laminectomy segments and discectomy segments between the tranexamic acid and control groups. The intraoperative blood loss (455.9 ± 206.6 ml vs 580.6 ± 224.3 ml, *p* < 0.05) and total blood loss (675.3 ± 170.3 ml vs 936.8 ± 306.4 ml, *p* < 0.01) in tranexamic acid group were significant lower than those in control group. The means of blood unit transfused (2.5 ± 1.0 vs 4.7 ± 2.4, *p* < 0.05) and Hb reduction in 48 h (22.5 ± 3.4 g/L vs 25.3 ± 3.9 g/L, *p* < 0.01) were significantly lower in tranexamic acid group than that in control group. There were no statistically significant differences in blood coagulation function pre-operation or 48 h post-operation between the tranexamic acid and the control groups. The requirements for patients to receive blood transfusion were fewer and durations of post-operational hospital stays were shorter in the tranexamic acid group, however, the difference did not achieve statistical significance. There was no significant difference in superficial or deep venous thrombosis of lower limbs or deterioration of neurological function between tranexamic acid group and control group.

**Conclusions:**

Application of intravenous tranexamic acid significantly reduces intraoperative and perioperative total blood loss without significant side effects in posterior operation of multilevel thoracic spine stenosis.

**Trial registration:**

At Chinese Clinal Trial Registry. http://www.chictr.org.cn/, ChiCTR2100054221. Registered on 11/12/2021.

**Supplementary Information:**

The online version contains supplementary material available at 10.1186/s12891-022-05361-2.

## Background

Massive intraoperative and postoperative blood loss occurs frequently and remains a challenge in multilevel thoracic spine stenosis (TSS) surgery. Extensive and rapid blood loss may lead to low blood pressure, which may lead to reduction of blood supply to the spinal cord and deterioration of neurological function. Allogenic blood transfusions can cause allergy, infection, acute kidney injury, et al. Both patients and government will face the problem of increased medical cost due to more usage of blood products, prolonged hospitalization, and complication managements. Some studies have found that tranexamic acid (TXA) is capable to reduce intraoperative and postoperative blood loss [1, 2]. TXA is a competitive inhibitor of plasmin and plasminogen and acts as an anti-fibrinolytic agent by competitively blocking lysine binding sites, thereby reducing blood loss [3]. Whether the application of TXA increases the risk of thrombotic events remains controversial, even several studies proved its efficacy of reducing the blood loss in complex spine surgery [4, 5]. But we found no study of usage of TXA had been carried out on operation of multilevel TSS, which is always accompanied by massive intraoperative bleeding.

This prospective randomized controlled trial was aimed to evaluate the efficacy and safety of intravenous usage of TXA in patients with posterior operation of multilevel TSS.

## Patients and Methods

This is a single center prospective randomized controlled trial. The study was approved by Ethics Committee of Peking University International Hospita and registered at Chinese Clinal Trial Registry on 11/12/2021 (No. ChiCTR2100054221, http://www.chictr.org.cn/). Written informed consents were obtained from all patients or their accompanying family members/guardians prior to their enrollments. A total of 68 patients with an average age of 56.5 ± 11.8(25 ~ 78) years were enrolled from October 2018 to August 2021. All patients were diagnosed with thoracic spinal stenosis according to symptoms and radiographic findings of thoracic spinal cord compression. The patients met with the inclusion criteria were selected into the trial, whereas, the patients met with the exclusion criteria were excluded. The inclusion and exclusion criteria are listed below:

- Inclusion criteria: 1. CT/MRI confirmed the TSS involved at least three segments (four vertebrae); 2. conservative treatment was ineffective and agreement of surgical treatment was obtained from the patient. 3. American Society of Anesthesiologists physical status class I, II, or III.
- Exclusion criteria: 1. patients combined with a coagulation or hemoglobin (Hb) disorder; 2. patients with hypo-hemoglobin (male < 120 g/L, female < 110 g/L); 3. patients using anticoagulant or antiplatelet drugs two weeks before randomization; 4. patients with renal insufficiency, history of thromboembolic event or significant cardiovascular disease; 5. allergy to TXA.

Patients were randomly divided into the two following groups by computer-based random number generation technique:

1. TXA group: receiving 15 mg/kg body weight at 15 min before skin incision and 1 mg/kg body weight per hour intravenously during operation until surgical site wound closure.
2. Control group: receiving the same dose of placebo (0.9% sodium chloride solution) intravenously during the same period.

All the operations were performed by the same three surgeons with prone position under general anesthesia. Exposed surgical site by separation of paravertebral muscles and removal of spinous process. Pedicle screw instrumentation, laminectomy, partial resection of the facet were performed on all patients over at least three segments. Then selected one or two segments discectomy and cages insertion were performed if necessary. Closed the wound layer by layer after placing one negative pressure drainage tube.

Intraoperative blood loss (IOBL) was calculated as: the weight of surgical sponges and gauze pieces—the weight of dry sponges and gauze pieces + the volume of suction canisters. (Note: 1 ml blood approximately weighs 1 g.)

Total blood loss (TBL) was the gross blood losses since operation initiation until 72 h post operation and calculated using the Gross formula [6]:

$$\mathrm{Total}\;\mathrm{blood}\;\mathrm{loss}\:=\:\mathrm{PBV}\;\times({\mathrm{Hct}}_{\mathrm{pre}}-{\mathrm{Hct}}_{\mathrm{post}})/{\mathrm{Hct}}_{\mathrm{ave}}$$

PBV: patient’s blood volume, Hct: hematocrit, Hct_pre_: preoperative hematocrit, Hct_post_: 72 h postoperative hematocrit; Hct_ave_: average of Hct_pre_ and Hct_post_.

PBV was calculated using the Nadler formula [7]:

$$\mathrm{PBV}\:=\:{\mathrm k}_1\;\times\mathrm{height}{(\mathrm m)}^3+{\mathrm k}_2\;\times\mathrm{weight}(\mathrm{kg})+{\mathrm k}_3$$

k_1_ = 0.3669, k_2_ = 0.03219, and k_3_ = 0.6041 for men, and k_1_ = 0.3561, k_2_ = 0.03308, and k_3_ = 0.1833 for women.

The same blood transfusion guidelines were used for all patients. Allogeneic blood transfusion was performed if Hb decreased less than 70.0 g/L or patients presented with obvious anemic symptoms.

Ultrasonography of both lower limbs was routinely performed 5 days after operation to screen for superficial or deep venous thrombosis. The examination may also be advanced or repeated if obvious symptoms, such as obvious lower limbs swelling, dyspnea, et al., were observed.

Baseline characteristics of each patient were documented and analyzed between groups, including age, gender, body mass index (BMI), ASA physical status, preoperative Hb, preoperative platelet (PLT), pathology required surgery, operation time, laminectomy segments, discectomy segments. Coagulation function indexes before and 48 h post-operation were recorded, including prothrombin time (PT), activated partial thromboplastin time (APTT), fibrinogen (FIB) and D-dimer. The IOBL, TBL, need and amount for blood transfusion, Hb reduction in 48 h, length of hospital stays after operation, superficial or deep venous thrombosis (DVT), cardiovascular or cerebrovascular events, pulmonary embolism (PE) and deterioration of neurological function were compared between groups.

SPSS 19.0 software (SPSS Inc., USA) was used for data analyses. The continuous variable was expressed as mean ± standard deviation, and independent sample t test or variance analysis (ANOVA) was used. Chi square test was adapted to analyze the categorical variable. Significant difference was defined as *p* < 0.05.

## Results

There was no significant difference in age, gender, BMI, ASA physical status, preoperative Hb, preoperative PLT, pathology required surgery, laminectomy segments, discectomy segments or operation time between groups (as shown in Table 1).

**Table 1:** Basic information of patients

	**TXA Group (*n* = 34)**	**Control Group (*n* = 34)**	***p* value**
Age (years)	54.8 ± 11.8	58.3 ± 11.6	0.222
Gender			0.079
Male	13	19	
Female	21	15	
BMI (kg/m^2^)	25.9 ± 3.6	27.1 ± 3.6	0.176
ASA status			0.735
I	14	17	
II	17	15	
III	3	2	
Preoperative Hb (g/L)	128.0 ± 11.6	132.1 ± 8.8	0.107
Preoperative PLT (10^9^/L)	288.4 ± 34.3	276.7 ± 34.4	0.162
Pathology			0.327
OLF	12	17	
OPLL	0	0	
OLF + OPLL	22	17	
Laminectomy segments	4.9 ± 1.6	4.8 ± 1.8	0.831
Discectomy segments	0.6 ± 0.7	0.5 ± 0.7	0.583
Operation time (min)	197.1 ± 79.0	210.0 ± 79.2	0.502

*TXA* Tranexamic Acid, *BMI*body mass index, *ASA*American Society of Anesthesiology, *Hb*hemoglobin, OLF  ossification of ligamentum flavum, *OPLL*ossification of posterior longitudinal ligament, *PLT*platelet

The IOBL, TBL, mean number of blood unit transfused and Hb reduction in 48 h between the two groups were significantly different. The need for blood transfusion was fewer and length of hospital stay after operation was shorter of TXA group than the control group, but didn’t reach statistical significance. The results demonstrate that the usage of TXA could reduce blood loss and blood transfusion amount, which may shorten the length of hospital stay (as shown in Table 2).

**Table 2:** Intraoperative and total blood loss

	**TXA Group**	**Control Group**	***p* value**
Intraoperative blood loss (ml)	455.9 ± 206.6	580.6 ± 224.3	0.020
Total blood loss (ml)	675.3 ± 170.3	936.8 ± 306.4	< 0.001
48 h Hb reduction (g/L)	22.5 ± 3.4	25.3 ± 3.9	0.003
Need for blood transfusion	4	9	0.123
Mean number of blood unit transfused	2.5 ± 1.0	4.7 ± 2.4	0.045
Hospital stay after operation (d)	8.5 ± 2.9	10.3 ± 4.5	0.060

*TXA* Tranexamic Acid, *Hb *hemoglobin

No significant difference was found in preoperative baseline coagulation function indexes and 48 h post operation between the two groups, which confirmed that the administration of TXA didn’t alter patient’s blood coagulation function (as shown in Table 3).

**Table 3:** Preoperative and postoperative coagulation function parameters

	**TXA Group**	**Control Group**	***p* value**
pre-op PT (s)	10. 5 ± 1.0	10.8 ± 0.9	0.174
pre-op APTT (s)	33.9 ± 3.4	32.4 ± 3.5	0.088
pre-op FIB (mg/dl)	294.4 ± 53.0	306.1 ± 53.2	0.368
pre-op D-dimer (ng/ml)	182.4 ± 35.3	170.7 ± 40.2	0.207
48 h post-op PT (s)	10.9 ± 0.9	11.2 ± 0.8	0.129
48 h post-op APTT (s)	33.2 ± 4.1	32.0 ± 3.4	0.162
48 h post-op FIB (mg/dl)	323.2 ± 63.5	304.4 ± 64.3	0.230
48 h post-op D-dimer (ng/ml)	185.1 ± 52.7	193.1 ± 36.9	0.473

*TXA* Tranexamic Acid, *PT*prothrombin time, *APTT*activated partial thromboplastin time, *FIB*fibrinogen

Moreover, there were no cardiovascular events, cerebrovascular events or PE happened in either group. There was no significant difference in superficial or deep venous thrombosis of lower limbs or deterioration of neurological function between TXA group and control group (as shown in Table 4).

**Table 4:** Possible complications associated with TXA and blood transfusion

	**TXA Group**	**Control Group**	***p* value**
Deep venous thrombosis	1	1	0.174
Superficial venous thrombosis	3	2	0.642
Cerebrovascular events	0	0	-
Cardiovascular events	0	0	-
Pulmonary embolism	0	0	-
Postoperative epilepsy	0	0	-
Renal damage	0	0	-
Visual perception of color	0	0	-
Allergic reaction	0	0	-
Deterioration of neurological function	2	3	0.642

*TXA* Tranexamic Acid

## Discussions

Besides the cardiac surgery, enhanced fibrinolysis had also been suggested to be a contributing factor to blood loss during spine surgery. Therefore, TXA as a counter fibrinolysis agent would play an important beneficial role in reducing blood loss. Bosch et al. demonstrated that TXA usage could diminished fibrinolysis as measured by fibrinolysis score and lysis percent [8]. Included on the list of the World Health Organization (WHO) List of Essential Medicines, TXA has taken its place as a widely used hemostatic agent in the clinical setting [9, 10].

Efficacy of TXA administered through different routes in various spine surgery has been researched in previous studies [11, 12]. However, there was no relevant study on multilevel TSS operation. Posterior laminectomy, instrumentation and selective discectomy are usually accompanied by massive bleeding intraoperation and post-operation due to decorticated bony surfaces, spongy vertebrae with rich blood supply and fragile venous plexus, which couldn’t be addressed by standard hemostatic methods [13, 14]. Whether TXA is safe and effective in surgery treatment on multilevel TSS remains to be addressed. Till date this study is the first randomized controlled trial focusing on the efficacy and safety of intravenous TXA in patients with posterior operation of multilevel TSS.

In this study, the usage of TXA could significantly reduce the IOBL, TBL and Hb reduction in 48 h. This result is consistent with the majority of previous literatures. Wang et al. [15] carried out a randomized controlled trial including 60 patients to assess the TXA usage in posterior approach of lumbar surgery, found that TXA could significantly reduce total blood loss as compared to control group (1260.7 ± 99.4 ml vs 1096.3 ± 85.0 ml, *p* < 0.01). In a retrospective study including 132 patients undergoing multilevel posterior spinal segmental instrumented fusion (≥ 5 levels), Choi et al. [16] found that the intraoperative estimated blood loss was significantly lower in TXA group than non-TXA group (841.01 ± 559.55 ml vs 1336.05 ± 923.36 ml, *p* = 0.002). In a meta-analysis of nine studies enrolling 713 patients about TXA usage in posterior lumbar fusion surgery, TXA could significantly decrease TBL, IOBL, postoperative blood loss and 24 h postoperative Hb decline [17].

Rather, some literatures have challenged the efficacy of TXA. Peter et al. found no significant difference in intraoperative blood loss or transfusion requirement between TXA and control group in patients with spinal deformity [18]. TXA did not significantly reduce transfusion requirement in Colomina’s study [19]. Bednar et al. found that TXA could not reduce estimated operative blood loss in patients with spine metastatic spine tumors [20]. These different voices remind us that we should conduct more rigorous high-level evident researches to better define the indications and dosage regimen of TXA.

Allogeneic blood transfusion is the most rapid and effective way to correct anemia when massive blood loss encountered. But this transfusion carries additional risks, including hemolytic transfusion reaction, transfusion related acute lung injury, infection transmission and immunoregulation effect [21]. In a retrospective study conducted by Xue et al. [22], TXA could significantly reduce intraoperative blood transfusion volumes in TSS surgery (963.64 ± 341.63 ml vs 1680.00 ± 442.01 ml, *p* = 0.01). Elwatidy et al. reported the blood loss during spine surgery in TXA group was 49% reduction than placebo group. Consequently, the amount of blood transfusion was 80% less in TXA than in placebo group [23]. Xu et al. [24] found that TXA usage in posterior lumbar interbody fusion surgery could significant decrease TBL and hidden blood loss, thus reducing the patients’ number of needing blood transfusion (12/30 vs 5/30, *p* < 0.05). In a randomized controlled trial meta-analysis conducted by Li et al. [25], the number of patients requiring transfusion was 27.4% (57/208) treated with TXA compared with 38.4% (78/203) treated with placebo (*p* = 0.01).

In our study, the number of patients who need blood transfusion is fewer in TXA group, but didn’t reach statistical significance. This might be caused by the large amount of blood loss, which always encountered in multilevel TSS surgery. But the usage of TXA could significantly reduce the amount of blood transfusion, therefore reducing the risk of blood transfusion complications, enhancing postoperative recovery, shortening the length of hospitalization, and reducing the financial cost [26].

A few studies revealed that maybe only the high dose TXA was more effective than placebo in reducing blood transfusion. A meta-analysis carried out by Yuan et al. which included 30 randomized controlled trial revealed high dose TXA could significantly reduce the amount of blood transfusion and the proportion of patients who needed transfusion when compared to low dose TXA or placebo [27].

The major concern surrounding the use of TXA and other antifibrinolytics is the potential for an increased risk of thrombotic events. Wangderman et al. retrospectively analyzed 266 patients undergoing lumbar and/or thoracic fusion surgery receiving intravenous TXA and followed 6 weeks after surgery. Total 5(1.9%) cases of DVT and 4(1.5%) case of PE were found [28]. Theoretically, the use of TXA may potentially increase the risk of thrombosis. However, many studies have confirmed that the use of TXA does not increase the risk of thrombotic complications, particularly DVT and PE [29].

Ko et al. retrospectively analyzed 122 patients who underwent lumbar fusion for degenerative spinal disease and received intravenous TXA. Only one case (0.8%) of DVT was confirmed by computed tomography angiography [30]. Eleven articles were included in a meta-analysis for TXA usage in spine surgery by Cheriyan et al. No DVT or PE was reported in TXA group [31].

High dose regimen may be more effective in reducing blood loss without increasing thrombosis risk. Only 2 DVTs and 1 PE happened in a retrospective study including 100 spine deformity patients. Moreover, there was no case of myocardial infarction, seizure, stroke, or acute renal failure [32]. Kim et al. [33] and Pernik et al. [34] reached similar results that no major complications reported even in high dose TXA group. TXA usage is safe even though multilevel spine surgery is risk factor of thromboembolism according to Goz et al. [35].

We found no statistical significance in PT, APTT, FIB, D-dimer preoperation or postoperation between groups, which indicated that TXA did not affect patient’s early coagulation function. The use of TXA will not change the blood into a hypercoagulable status which might lead to thrombotic events.

We reported no other potential adverse effects including postoperative epilepsy, renal damage, visual perception of color or allergic reaction et al. Similarly, these side effects were uncommon in previous literatures [36, 37]. Doctors should be alert to such side effects, but not over feared. It must be noted that many studies excluded patients with history of cardiovascular disease, thromboembolic events, et al. Therefore, the safety of TXA in patients with high-risk of thrombosis needs to be further evaluated.

The limitations of this study included its small sample size and single center design. This study only compared the difference between intravenous administration of TXA and placebo. In the future, more trials can be carried out to verify the efficacy and safety of local and oral administration of TXA, which might also play an effective role especially in reducing postoperative blood loss [38]. Due to there is no unified standard, the dose of TXA used in our study was based on other studies [39] and our clinical experience, dose selection bias might exist. We didn’t analyze the efficacy and safety of different doses because of relatively small sample size. The patients included in this study were relatively low-risk patients.

## Conclusions

Intravenous TXA significantly reduces intraoperative and perioperative total blood loss, amount of blood transfusion and Hb reduction for posterior operation of multilevel TSS. TXA doesn’t affect patient coagulation function nor increase the risk of thrombosis events postoperatively. In summary, usage of intravenous TXA can be an effective and safe way to reduce blood loss and blood transfusion in posterior operation of multilevel TSS.

## Supplementary Information

**Additional file 1.** Data.sav

## Data Availability

All data analyzed during this study are included in this published article and its supplementary information files.

## Abbreviations

TXA: Tranexamic acid
TSS: Thoracic spine stenosis
TBL: Total blood loss
IOBL: Intraoperative blood loss
PBV: Patient’s blood volume
Hct: Hematocrit
BMI: Body mass index
Hb: Hemoglobin
PLT: Platelet
PT: Prothrombin time
APTT: Activated partial thromboplastin time
FIB: Fibrinogen
DVT: Deep venous thrombosis
PE: Pulmonary embolism
ASA: American Society of Anesthesiology
OLF: Ossification of ligamentum flavum
OPLL: Ossification of posterior longitudinal ligament
