# Supplementary material for: The efficacy and safety of intravenous tranexamic acid in patients with posterior operation of multilevel thoracic spine stenosis: a prospective randomized controlled trial
Source: BMC Musculoskelet Disord. 2022 May 2;23:410. doi: 10.1186/s12891-022-05361-2 (PMC9063045; doi:10.1186/s12891-022-05361-2)
Supplement: Supplementary file 1 — Additional file 1. Data.sav [file 12891_2022_5361_MOESM1_ESM.pdf]

data.sav

	group	gender	age	BMI	preHb	prePLT
1	1.00	1.00	31.00	24.30	131.00	300.00
2	1.00	0.00	69.00	20.80	127.00	315.00
3	1.00	0.00	52.00	30.10	123.00	317.00
4	1.00	0.00	63.00	25.40	124.00	265.00
5	1.00	0.00	42.00	20.70	146.00	266.00
6	1.00	0.00	56.00	28.40	120.00	310.00
7	1.00	0.00	56.00	21.70	119.00	247.00
8	1.00	0.00	66.00	22.20	100.00	210.00
9	1.00	0.00	71.00	26.00	133.00	236.00
10	1.00	1.00	72.00	27.20	125.00	313.00
11	1.00	0.00	56.00	31.80	126.00	267.00
12	1.00	0.00	47.00	26.40	142.00	282.00
13	1.00	0.00	48.00	30.60	131.00	318.00
14	1.00	1.00	55.00	29.30	122.00	346.00
15	1.00	1.00	51.00	23.90	143.00	200.00
16	1.00	0.00	43.00	20.80	146.00	295.00
17	1.00	1.00	47.00	21.70	131.00	281.00
18	1.00	1.00	64.00	20.50	121.00	237.00
19	1.00	1.00	59.00	28.20	137.00	316.00
20	1.00	0.00	45.00	23.30	118.00	293.00
21	1.00	0.00	73.00	24.50	134.00	292.00
22	1.00	0.00	51.00	30.50	120.00	328.00
23	1.00	1.00	25.00	34.00	123.00	302.00
24	1.00	0.00	67.00	25.90	100.00	286.00
25	1.00	1.00	41.00	22.10	137.00	310.00
26	1.00	0.00	74.00	26.30	129.00	280.00
27	1.00	1.00	53.00	21.10	145.00	298.00
28	1.00	1.00	61.00	28.80	138.00	302.00
29	1.00	1.00	61.00	24.70	118.00	258.00
30	1.00	0.00	52.00	26.00	142.00	332.00
31	1.00	0.00	44.00	29.00	129.00	305.00
32	1.00	0.00	43.00	30.30	113.00	272.00
33	1.00	1.00	62.00	26.50	139.00	339.00
34	1.00	0.00	63.00	26.70	121.00	289.00
35	0.00	1.00	64.00	29.10	130.00	253.00
36	0.00	0.00	47.00	29.90	142.00	254.00
37	0.00	1.00	58.00	21.70	124.00	276.00

	time	lamlevel	disclevel	OBL	TBL	transfusion
1	240.00	3.00	1.00	840.00	590.00	0.00
2	240.00	3.00	0.00	620.00	900.00	0.00
3	120.00	6.00	1.00	570.00	790.00	0.00
4	210.00	5.00	1.00	810.00	780.00	1.00
5	100.00	6.00	1.00	520.00	530.00	0.00
6	230.00	5.00	0.00	410.00	520.00	0.00
7	90.00	3.00	0.00	470.00	670.00	1.00
8	110.00	5.00	0.00	620.00	680.00	0.00
9	100.00	3.00	0.00	90.00	940.00	0.00
10	260.00	7.00	2.00	420.00	480.00	0.00
11	260.00	6.00	1.00	650.00	950.00	0.00
12	120.00	3.00	0.00	110.00	350.00	0.00
13	300.00	5.00	0.00	660.00	870.00	1.00
14	270.00	8.00	1.00	700.00	560.00	1.00
15	160.00	3.00	1.00	660.00	460.00	0.00
16	90.00	3.00	0.00	630.00	420.00	0.00
17	260.00	4.00	1.00	120.00	810.00	0.00
18	290.00	6.00	0.00	430.00	700.00	0.00
19	190.00	4.00	0.00	570.00	460.00	0.00
20	280.00	4.00	1.00	560.00	370.00	0.00
21	120.00	4.00	0.00	580.00	730.00	0.00
22	110.00	6.00	1.00	120.00	750.00	0.00
23	170.00	5.00	0.00	450.00	930.00	0.00
24	160.00	7.00	1.00	390.00	780.00	0.00
25	200.00	3.00	1.00	210.00	690.00	0.00
26	220.00	3.00	0.00	230.00	780.00	0.00
27	320.00	4.00	1.00	500.00	720.00	0.00
28	100.00	4.00	0.00	500.00	850.00	0.00
29	400.00	8.00	2.00	380.00	590.00	0.00
30	120.00	5.00	2.00	150.00	660.00	0.00
31	280.00	6.00	0.00	530.00	750.00	0.00
32	190.00	8.00	1.00	130.00	660.00	0.00
33	190.00	5.00	0.00	440.00	430.00	0.00
34	200.00	5.00	0.00	430.00	810.00	0.00
35	220.00	8.00	0.00	260.00	980.00	0.00
36	290.00	7.00	0.00	440.00	480.00	0.00
37	200.00	5.00	0.00	470.00	490.00	0.00

	amount	PT	APTT	FIB	Ddimer	PT48
1	.	11.50	38.40	327.00	197.00	11.10
2	.	9.70	34.90	390.00	230.00	11.40
3	.	9.70	34.10	233.00	185.00	9.80
4	2.00	9.80	31.20	384.00	167.00	10.50
5	.	11.30	35.40	274.00	125.00	11.40
6	.	9.00	26.30	219.00	223.00	10.70
7	4.00	10.10	28.90	363.00	202.00	11.10
8	.	10.70	35.20	349.00	215.00	12.30
9	.	10.90	34.60	312.00	215.00	10.20
10	.	9.80	32.30	210.00	190.00	9.40
11	.	9.90	35.40	286.00	135.00	12.30
12	.	10.40	38.40	305.00	219.00	11.20
13	2.00	9.40	28.70	301.00	142.00	11.80
14	2.00	12.10	38.40	371.00	160.00	11.00
15	.	9.00	28.90	371.00	218.00	11.90
16	.	13.00	34.60	255.00	203.00	12.40
17	.	9.60	36.40	278.00	215.00	9.70
18	.	11.20	38.40	295.00	163.00	9.40
19	.	9.60	35.90	289.00	148.00	9.50
20	.	9.40	32.80	305.00	215.00	10.70
21	.	11.70	36.10	301.00	125.00	10.60
22	.	10.90	28.50	333.00	129.00	9.40
23	.	10.40	38.40	211.00	228.00	11.40
24	.	9.90	36.90	228.00	215.00	10.80
25	.	9.70	29.70	317.00	218.00	10.40
26	.	11.00	34.90	330.00	146.00	10.80
27	.	10.50	35.10	202.00	128.00	9.60
28	.	10.70	29.10	252.00	233.00	12.00
29	.	9.60	33.10	260.00	175.00	10.60
30	.	9.60	30.30	292.00	173.00	10.80
31	.	11.80	36.20	319.00	187.00	11.00
32	.	11.30	37.50	354.00	169.00	12.40
33	.	12.20	34.80	229.00	141.00	12.10
34	.	11.80	32.70	266.00	166.00	10.70
35	.	12.30	35.10	362.00	121.00	11.50
36	.	11.90	33.50	344.00	189.00	11.20
37	.	12.40	37.90	208.00	153.00	10.50

	APTT48	FIB48	Ddimer48	DVT	SVT	SCI
1	35.00	313.00	165.00	0.00	0.00	0.00
2	33.20	227.00	215.00	0.00	0.00	0.00
3	28.00	398.00	167.00	0.00	1.00	0.00
4	26.20	380.00	207.00	0.00	1.00	1.00
5	34.70	287.00	178.00	0.00	1.00	0.00
6	40.00	373.00	148.00	1.00	0.00	0.00
7	35.60	284.00	210.00	0.00	0.00	0.00
8	37.50	415.00	158.00	0.00	0.00	0.00
9	31.00	265.00	175.00	0.00	0.00	0.00
10	40.00	415.00	178.00	0.00	0.00	0.00
11	29.20	317.00	186.00	0.00	0.00	1.00
12	34.70	415.00	161.00	0.00	0.00	0.00
13	30.00	234.00	110.00	0.00	0.00	0.00
14	36.70	357.00	175.00	0.00	0.00	0.00
15	40.00	380.00	194.00	0.00	0.00	0.00
16	35.90	321.00	237.00	0.00	0.00	0.00
17	25.80	280.00	132.00	0.00	0.00	0.00
18	29.70	201.00	188.00	0.00	0.00	0.00
19	29.50	312.00	222.00	0.00	0.00	0.00
20	33.30	361.00	221.00	0.00	0.00	0.00
21	33.00	415.00	200.00	0.00	0.00	0.00
22	40.00	260.00	129.00	0.00	0.00	0.00
23	28.10	252.00	200.00	0.00	0.00	0.00
24	34.30	296.00	400.00	0.00	0.00	0.00
25	30.40	334.00	232.00	0.00	0.00	0.00
26	34.10	256.00	130.00	0.00	0.00	0.00
27	35.20	395.00	126.00	0.00	0.00	0.00
28	33.60	415.00	222.00	0.00	0.00	0.00
29	38.20	279.00	183.00	0.00	0.00	0.00
30	32.50	376.00	216.00	0.00	0.00	0.00
31	29.90	230.00	130.00	0.00	0.00	0.00
32	25.90	303.00	235.00	0.00	0.00	0.00
33	36.20	321.00	137.00	0.00	0.00	0.00
34	32.60	320.00	126.00	0.00	0.00	0.00
35	29.00	284.00	166.00	0.00	0.00	0.00
36	36.60	244.00	138.00	0.00	0.00	0.00
37	37.30	304.00	179.00	0.00	0.00	0.00

	hostime
1	20.00
2	6.00
3	8.00
4	7.00
5	7.00
6	11.00
7	7.00
8	7.00
9	8.00
10	7.00
11	7.00
12	14.00
13	6.00
14	9.00
15	9.00
16	8.00
17	9.00
18	16.00
19	9.00
20	8.00
21	6.00
22	9.00
23	9.00
24	8.00
25	8.00
26	7.00
27	5.00
28	7.00
29	8.00
30	8.00
31	9.00
32	8.00
33	6.00
34	8.00
35	8.00
36	8.00
37	8.00

	group	gender	age	BMI	preHb	prePLT
38	0.00	0.00	77.00	30.40	137.00	315.00
39	0.00	1.00	52.00	26.80	130.00	285.00
40	0.00	1.00	74.00	21.10	142.00	251.00
41	0.00	1.00	52.00	31.20	130.00	322.00
42	0.00	1.00	51.00	26.30	132.00	236.00
43	0.00	0.00	62.00	21.90	123.00	237.00
44	0.00	1.00	64.00	29.40	119.00	291.00
45	0.00	1.00	50.00	28.90	141.00	283.00
46	0.00	1.00	46.00	31.20	133.00	242.00
47	0.00	0.00	44.00	30.60	118.00	318.00
48	0.00	0.00	64.00	23.50	136.00	267.00
49	0.00	0.00	78.00	28.10	126.00	317.00
50	0.00	0.00	49.00	27.50	143.00	261.00
51	0.00	0.00	53.00	29.50	146.00	284.00
52	0.00	1.00	68.00	29.50	122.00	241.00
53	0.00	0.00	56.00	27.10	124.00	326.00
54	0.00	1.00	60.00	27.00	131.00	340.00
55	0.00	1.00	59.00	31.10	142.00	311.00
56	0.00	0.00	66.00	20.40	140.00	271.00
57	0.00	1.00	28.00	23.60	135.00	254.00
58	0.00	0.00	73.00	25.70	126.00	262.00
59	0.00	0.00	52.00	21.20	142.00	278.00
60	0.00	1.00	63.00	19.50	144.00	271.00
61	0.00	1.00	76.00	31.10	119.00	327.00
62	0.00	1.00	61.00	24.70	127.00	288.00
63	0.00	1.00	46.00	27.20	122.00	287.00
64	0.00	0.00	47.00	33.90	127.00	200.00
65	0.00	0.00	45.00	29.60	136.00	200.00
66	0.00	0.00	57.00	27.60	141.00	293.00
67	0.00	1.00	78.00	26.80	144.00	301.00
68	0.00	1.00	62.00	27.70	118.00	264.00

	time	lamlevel	disclevel	OBL	TBL	transfusion
38	150.00	3.00	0.00	500.00	1340.00	0.00
39	180.00	6.00	1.00	800.00	840.00	0.00
40	180.00	8.00	1.00	810.00	1340.00	1.00
41	210.00	7.00	0.00	440.00	500.00	0.00
42	320.00	6.00	0.00	630.00	600.00	0.00
43	220.00	4.00	1.00	510.00	1130.00	0.00
44	270.00	3.00	1.00	900.00	740.00	0.00
45	100.00	7.00	0.00	490.00	540.00	0.00
46	210.00	3.00	0.00	110.00	680.00	0.00
47	200.00	3.00	0.00	620.00	480.00	0.00
48	180.00	7.00	2.00	450.00	1020.00	0.00
49	130.00	4.00	0.00	620.00	1340.00	1.00
50	230.00	3.00	1.00	200.00	1210.00	1.00
51	170.00	5.00	0.00	730.00	1290.00	0.00
52	140.00	3.00	1.00	860.00	740.00	1.00
53	280.00	4.00	0.00	450.00	750.00	0.00
54	130.00	4.00	0.00	670.00	1000.00	0.00
55	420.00	7.00	1.00	440.00	1270.00	0.00
56	260.00	3.00	0.00	810.00	1020.00	1.00
57	150.00	3.00	0.00	520.00	540.00	0.00
58	160.00	3.00	2.00	1100.00	1220.00	1.00
59	260.00	3.00	0.00	790.00	1340.00	0.00
60	280.00	4.00	0.00	200.00	1090.00	0.00
61	180.00	4.00	1.00	500.00	780.00	0.00
62	150.00	4.00	0.00	460.00	720.00	1.00
63	170.00	3.00	0.00	960.00	1140.00	1.00
64	100.00	4.00	1.00	570.00	670.00	0.00
65	430.00	8.00	2.00	530.00	860.00	0.00
66	120.00	3.00	0.00	460.00	1330.00	1.00
67	280.00	6.00	1.00	800.00	990.00	0.00
68	170.00	7.00	1.00	640.00	1390.00	0.00

	amount	PT	APTT	FIB	Ddimer	PT48
38	.	10.00	27.70	323.00	171.00	11.30
39	.	10.20	26.50	330.00	229.00	9.70
40	4.00	10.90	29.70	325.00	235.00	9.70
41	.	9.40	37.70	379.00	117.00	11.70
42	.	10.00	36.60	312.00	182.00	11.70
43	.	10.90	32.60	275.00	162.00	10.20
44	.	10.30	33.80	352.00	139.00	11.40
45	.	10.00	35.30	320.00	132.00	10.70
46	.	11.20	37.80	348.00	153.00	12.20
47	.	11.50	30.50	315.00	133.00	10.80
48	.	9.90	34.50	373.00	134.00	11.40
49	4.00	9.70	32.00	223.00	224.00	12.30
50	2.00	9.50	31.70	324.00	210.00	10.80
51	.	11.50	32.00	203.00	214.00	11.00
52	6.00	10.00	30.00	300.00	201.00	11.90
53	.	10.20	36.60	351.00	137.00	9.40
54	.	11.20	29.20	322.00	173.00	10.70
55	.	9.90	29.50	388.00	169.00	10.00
56	2.00	10.40	34.50	292.00	238.00	12.30
57	.	9.50	32.10	235.00	113.00	12.30
58	6.00	10.70	32.10	203.00	166.00	11.70
59	.	12.40	36.60	313.00	194.00	11.30
60	.	10.60	35.20	259.00	225.00	11.50
61	.	11.20	31.00	250.00	117.00	11.90
62	8.00	12.20	36.50	274.00	151.00	10.40
63	2.00	10.20	28.80	312.00	117.00	12.20
64	.	11.30	28.90	398.00	135.00	11.50
65	.	11.90	32.60	318.00	140.00	11.30
66	8.00	11.90	33.20	290.00	181.00	11.60
67	.	12.40	25.60	341.00	232.00	10.90
68	.	10.70	25.90	246.00	216.00	12.40

	APTT48	FIB48	Ddimer48	DVT	SVT	SCI
38	33.30	248.00	191.00	0.00	0.00	0.00
39	31.80	285.00	236.00	0.00	0.00	0.00
40	32.10	222.00	228.00	0.00	0.00	0.00
41	27.60	275.00	113.00	0.00	0.00	0.00
42	34.50	395.00	185.00	0.00	0.00	0.00
43	32.40	213.00	135.00	0.00	0.00	0.00
44	25.80	284.00	228.00	0.00	0.00	0.00
45	26.00	228.00	227.00	0.00	0.00	0.00
46	31.30	391.00	194.00	0.00	0.00	0.00
47	35.00	304.00	153.00	0.00	0.00	0.00
48	27.40	210.00	178.00	0.00	0.00	0.00
49	30.60	250.00	206.00	0.00	0.00	0.00
50	34.90	260.00	150.00	0.00	0.00	0.00
51	32.90	229.00	207.00	0.00	0.00	0.00
52	30.50	328.00	164.00	0.00	0.00	0.00
53	31.40	325.00	203.00	0.00	0.00	0.00
54	40.00	388.00	150.00	0.00	0.00	0.00
55	31.10	249.00	250.00	0.00	0.00	1.00
56	31.50	312.00	167.00	0.00	0.00	0.00
57	31.10	270.00	165.00	0.00	0.00	0.00
58	25.60	361.00	203.00	0.00	0.00	0.00
59	31.20	382.00	230.00	0.00	0.00	0.00
60	32.00	258.00	192.00	0.00	0.00	1.00
61	32.60	383.00	250.00	0.00	0.00	0.00
62	37.50	337.00	226.00	0.00	1.00	0.00
63	33.50	386.00	158.00	0.00	1.00	1.00
64	31.10	267.00	250.00	0.00	0.00	0.00
65	32.20	415.00	177.00	1.00	0.00	0.00
66	30.00	415.00	207.00	0.00	0.00	0.00
67	28.90	264.00	208.00	0.00	0.00	0.00
68	37.10	383.00	250.00	0.00	0.00	0.00

	hostime
38	7.00
39	7.00
40	8.00
41	14.00
42	8.00
43	9.00
44	9.00
45	8.00
46	7.00
47	8.00
48	9.00
49	9.00
50	8.00
51	15.00
52	7.00
53	11.00
54	11.00
55	7.00
56	11.00
57	8.00
58	8.00
59	8.00
60	14.00
61	17.00
62	25.00
63	7.00
64	24.00
65	9.00
66	9.00
67	15.00
68	8.00
